# Erasing Synapses in Sleep: Is It Time to Be SHY?

**DOI:** 10.1155/2012/264378

**Published:** 2012-02-28

**Authors:** Marcos Gabriel Frank

**Affiliations:** Department of Neuroscience, Perelman School of Medicine, University of Pennsylvania, Philadelphia, PA 19104-6074, USA

## Abstract

Converging lines of evidence strongly support a role for sleep in brain plasticity. An elegant idea that may explain how sleep accomplishes this role is the “synaptic homeostasis hypothesis (SHY).” According to SHY, sleep promotes net synaptic weakening which offsets net synaptic strengthening that occurs during wakefulness. SHY is intuitively appealing because it relates the homeostatic regulation of sleep to an important function (synaptic plasticity). SHY has also received important experimental support from recent studies in *Drosophila melanogaster*. There remain, however, a number of unanswered questions about SHY. What is the cellular mechanism governing SHY? How does it fit with what we know about plasticity mechanisms in the brain? In this review, I discuss the evidence and theory of SHY in the context of what is known about Hebbian and non-Hebbian synaptic plasticity. I conclude that while SHY remains an elegant idea, the underlying mechanisms are mysterious and its functional significance unknown.

## 1. Introduction

**Figure 1:**
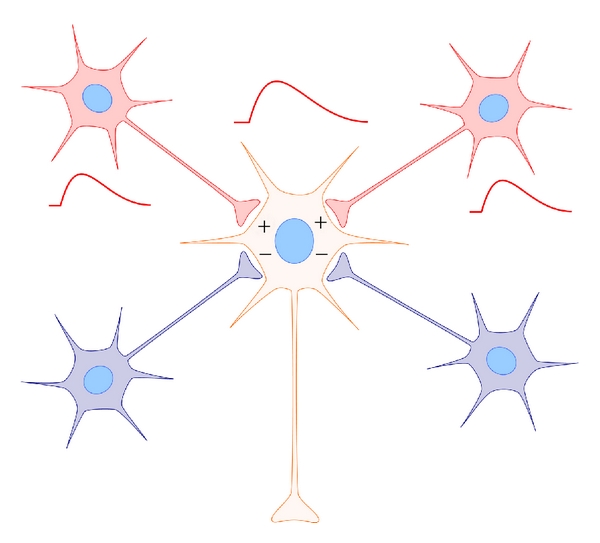
Hebbian plasticity. Classic Hebbian plasticity includes homosynaptic long-term synaptic potentiation (LTP) and long-term synaptic depression (LTD). Coincident activation in pre-synaptic neuronal inputs and the post-synaptic neuron strengthens specific synapses (shown in red). Inactive pre-synaptic inputs (or inputs out of phase with post-synaptic depolarization) are not potentiated and/or are depressed (shown in blue). The term “homosynaptic” refers to the fact that plasticity only occurs at the stimulated synapse.

**Figure 2:**
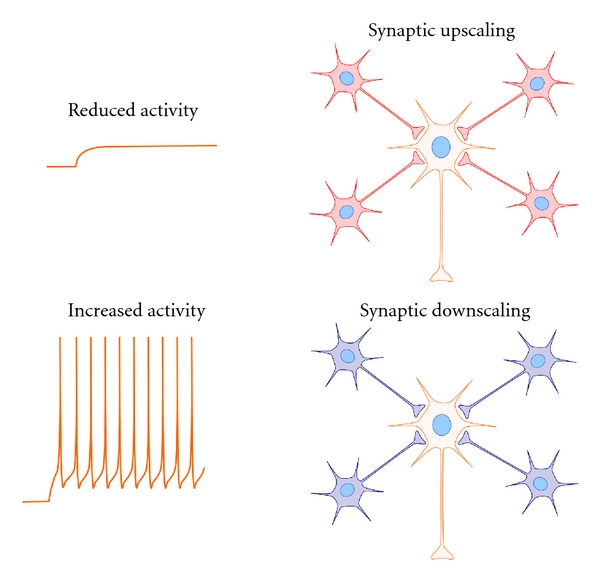
Synaptic scaling. Synaptic scaling involves global adjustments of all synapses in a neuron in response to global changes in neuronal activity. *Decreases* in neuronal activity lead to global increases in synaptic efficacy in the target neuron (upscaling). *Increases* in neuronal activity lead to global decreases in synaptic efficacy in the target neuron (downscaling). This form of plasticity is considered “non-Hebbian” because it involves global adjustments of synapses, rather than input-specific changes in discrete synapses.

A preponderance of evidence supports the view that sleep promotes brain plasticity. For example, a large number of studies in humans and animals show that sleep enhances and stabilizes memory (i.e., consolidation, reviewed in [1–3]). What remain more mysterious are the underlying cellular mechanisms that promote plastic changes in the sleeping brain. Until quite recently, the synaptic mechanisms were generally considered to be Hebbian. That is, scientists conceptualized and investigated the problem in terms of what was known about long-term synaptic potentiation (LTP) and depression (LTD) (reviewed in [4, 5]). These forms of plasticity are considered Hebbian because they involve changes in specific synapses mediated by coordinated activity in pre- and postsynaptic neurons (Figure 1). In the late 1990s, a non-Hebbian type of plasticity was described that adjusted all synapses in a neuron or network of neurons upward or downward in response to global changes in activity (reviewed in [6–8]). This type of plasticity was dubbed “synaptic scaling” or “homeostatic synaptic plasticity” (Figure 2), and as a concept was incorporated into the synaptic homeostasis hypothesis (SHY) [9–11].

According to SHY, sleep promotes global, or “net” synaptic downscaling which offsets global or “net” synaptic potentiation produced by wakefulness [9, 10]. The idea that sleep weakens synapses is not novel [4, 12, 13], but SHY has a number of unique aspects. First, it attempts to connect the homeostatic regulation of sleep to a putative function of sleep (plasticity). Sleep homeostasis refers to an enigmatic process that makes animals sleep longer (or more intensely) as a function of prior time awake [14]. It is logical that the regulation of sleep is linked to its core function [15], but the nature of this linkage has proven elusive [16]. Second, SHY is supported by an impressive number of findings in insects [17–19], rodents [20–22], and humans [23], mostly reported by the same group. These experiments use an equally impressive variety of tools including sophisticated molecular, cellular, electrophysiological, and computational techniques.

Despite the elegance of SHY and the arsenal of resources employed in its pursuit, there remain important unanswered questions about its core concepts, and the significance of its supportive findings. In this review, I take a closer look at SHY. I begin by briefly reviewing the basic concepts of synaptic scaling (see [6–8, 24] for more extensive discussion). I then review the theory of SHY and its empirical supports. I then address the proposed mechanisms governing SHY and the extent to which they agree with our current understanding of synaptic plasticity. In cases where they do not agree, alternative mechanisms are considered. I then discuss unanswered questions and future experiments that may provide strong tests of SHY.

## 2. Synaptic Scaling

Synaptic scaling refers to global adjustments of all synapses in a neuron or a neuronal network in response to global changes in activity. These adjustments manifest either as changes in synapse number or in post synaptic electrical currents (defined here as “synaptic efficacy”) [6–8, 24]. They are considered homeostatic because they restore total synaptic inputs to a specific range while maintaining the *relative *strength of all synapses. Synapses can be “downscaled” and “upscaled” which is thought to offset Hebbian changes that if left unchecked would quickly saturate synaptic strength in a network [6–8]. Because synaptic scaling involves global changes in synapses, rather than input-specific change at a given synapse, it is considered non-Hebbian. More recent work suggests that synaptic scaling can also occur regionally (i.e., “local” scaling) but since this is less understood, it is not discussed further here [6–8, 24]. In addition, there is increasing evidence that synaptic scaling also occurs in inhibitory circuits [6–8]. However, as inhibitory synapses do not factor prominently in SHY [9, 10], inhibitory scaling is also not discussed.

The central principle of synaptic scaling is quite simple: *decreases* in neuronal or network activity *upscale* synapses while *increases* in neuronal or network activity *downscale* synapses. This principle is key to our later discussion of the mechanisms of SHY. The first demonstration of synaptic scaling was made in cell culture where drugs that inhibited neuronal activity (e.g., tetrodotoxin) led to upscaling while drugs that increased neuronal activity (e.g., bicuculine) led to downscaling [6–8]. The effects of synaptic scaling manifested as changes in the frequency or amplitude of miniature excitatory postsynaptic currents (mEPSCs). More recent studies suggest that synaptic scaling also occurs *in vivo *under more naturalistic manipulations. For example, sensory deprivation *in vivo* leads to compensatory synaptic upscaling (reviewed in [6]) as measured by changes in dendrite spine morphology [25] and cortical mEPSCs [26]. In addition, although early *in vitro* studies suggested that scaling was a slow process (occurring over 24–48 hours [27]), more recent findings demonstrate that it can occur much more rapidly (over minutes [28]).

### 2.1. The Cellular Mechanisms of Synaptic Scaling

A very exciting area of neuroscience is the search for what distinguishes scaling mechanistically from classic Hebbian forms of plasticity like LTP. This understanding remains far from complete, but several important clues have been discovered. Calcium flux appears central to the initiation of synaptic scaling [6, 7]. In cortical neurons, decreases in intracellular calcium lead to upscaling, while increases lead to downscaling. These events are in turn mediated by calcium-/calmodulin- dependent kinases which remove or insert AMPA receptors into the plasma membrane [29]. Although Hebbian LTP and LTD also involve the trafficking of AMPAR this requires different calcium dynamics (opposite to those mediate upscaling and downscaling, resp.). Other signaling molecules linked to synaptic scaling are shown in Table 1. Some of these molecules and signaling pathways have also been examined across sleep and wakefulness (more detailed description of these mechanisms can be found in [7, 24]). Their pattern of expression will also be key to our discussion of SHY.

## 3. The Synaptic Homeostasis Hypothesis (SHY)

The central principle of SHY is also refreshingly simple as it embodies the basic concept of synaptic scaling and its importance into the sleep/wake cycle. Wakefulness is associated with net synaptic potentiation while sleep is associated with net synaptic downscaling. Although the term “net” is somewhat nebulous, this construction is useful because it divides the scaling problem neatly into two parts. The potentiation hypothesized to occur in wakefulness is considered Hebbian, because in the original description of SHY, analogies are drawn between molecular and cellular correlates of wakefulness and LTP. For example, as stated by Tononi:

“…the first part of the hypothesis states that wakefulness is generally accompanied by LTP-like changes in the brain…” and that molecular correlates of LTP are “restricted to wakefulness” ([9, page 144]). This proposition is retained in a second theoretical paper by Tononi:

“During wakefulness we interact with the environment and acquire information about it…the neuromodulatory milieu (e.g., high levels of noradrenaline, NA) favors the storage of information, which occurs largely through long-term potentiation of synaptic strength” ([10, page 50]).

With respect to synaptic downscaling, this is hypothesized to be driven by slow-wave electroencephalogram (EEG) activity (SWA) in non-REM sleep:

“According to the hypothesis, slow waves occurring in the cortex during sleep would actively promote a generalized depression or downscaling of synapses.” ([9, page 145]).

And “…slow waves are not just an epiphenomenon of the increased synaptic strength, but have a role to play. The repeated sequences of depolarization-hyperpolarization cause the downscaling of the synapses impinging on each neuron…” ([10, page 53]).

In contrast to the proposed Hebbian-like synaptic changes in wakefulness, how SWA downscales synapses is less precisely defined:

“…we hypothesize that downscaling is likely to use many of the same molecular mechanisms involved in depression/deprotentiation and activity-dependent scaling” ([10, page 54]).

This broad description of the mechanisms of downscaling has the advantage that *any* evidence of synaptic weakening after sleep, however measured, can be cited in support of the theory. It is disadvantageous in that no single, clear mechanism is presented for careful and in depth investigation. This is a limitation of SHY because it leaves supportive findings open to alternative explanations that may be unrelated to sleep.

## 4. The Theory of SHY Re-Examined

Let us consider the principal claims of SHY. First, it is argued that learning (a waking phenomenon) is largely mediated by LTP. Second, it is argued that the neurochemical and molecular milieu of wakefulness preferentially favors synaptic strengthening while sleep favors synaptic weakening.

### 4.1. Learning and LTP

Learning is a deceptively simple term for a complex set of neural events, often involving multiple brain areas and signaling pathways [43–45]. Perhaps not surprisingly, while some forms of learning may be associated with LTP [46, 47], others are not or involve a mixture of LTP- and LTD-like synaptic changes [48–51]. For example, extinction is a form of learning with obvious survival value to animals [52] as it allows them to change prior learned behaviors in light of new information. Several forms of extinction involve synaptic weakening, either through NMDA receptor mediated LTD [53] or endocannabinoid-mediated LTD [54]. A related phenomenon, the ability to reverse learning in order to adapt to new information (behavioral flexibility) requires LTD-like mechanisms (e.g., AMPA receptor internalization) [55]. Spatial memory also appears to require LTD and AMPAR internalization [56]. Exposure to novel environments induces (or involves) LTP-like synaptic changes in the hippocampus, *and* changes similar to LTD [48–50]. In perirhinal cortex, visual experience weakens responses to familiar visual stimuli, a phenomenon that may contribute to visual recognition memory. More specifically, LTD is prominent in perirhinal cortex, and peptides that block AMPA receptor internalization block both LTD and visual recognition memory [57, 58]. A requirement for LTD in some forms of learning is not restricted to vertebrates. Associative olfactory learning in the honey bee mushroom body involves a dampening of responses in PE1 neurons—which in turn may involve LTD or changes in inhibitory input [59]. To summarize, there are many forms of learning that require different types of synaptic plasticity. It is thus improbable that sleep need—to the extent this is determined by learning—is determined solely by Hebbian LTP (or any other single form of synaptic strengthening).

### 4.2. Sleep, Synaptic Strengthening, and Synaptic Weakening

SHY appears to have its origins in two molecular studies from the Tononi and Cirelli laboratory [11]. It was shown that a number of mRNAs implicated in LTP such as *bdnf*, *arc,* and *narp* were upregulated in the neocortex in animals sacrificed after the end of the normal wake period, or after an additional 6 hour sleep deprivation period which extended into the rest period. For *bdnf* and *arc*, the cortical expression of transcript and protein required noradrenaline (NA) as NA depletion reduced the expression of these molecules during wakefulness [30, 31]. It was then proposed that the comparatively low levels of NA during sleep ensured that “synaptic activity is not followed by synaptic potentiation” ([10, page 51]).

Before addressing this issue, two caveats should be discussed. First, there are relatively few plasticity-related molecules with single effects on synaptic efficacy. Molecules reported at higher levels in the neocortex after wakefulness relative to sleep mediate not only LTP, but also LTD, non-Hebbian scaling (e.g., Arc [30, 60]), and the synthesis of GABA in inhibitory interneurons (i.e., BDNF [61, 62]). Other molecules cited as evidence of synaptic weakening during sleep (e.g., CaMKIV [30]) are also required for some forms of LTP [63]. Therefore, the mere appearance of these molecules—especially if only measured at the transcript level—does not tell you if synapses are weakening or strengthening. Second, neuromodulators such as NA have complex and diverse effects on synaptic plasticity. NA, acetylcholine, and serotonin can promote LTP or LTD depending on brain location (or cortical lamina) and receptor subtypes [64–71]. Thus, the type and valence of plastic change cannot be predicted solely by the relative concentrations of these neuromodulators.

The cerebellum provides an illustrative example of this problem. The cerebellum exhibits state-dependent changes in mRNA transcript levels that are strikingly similar to those in the neocortex [30]. In the neocortex *and* cerebellum mRNA transcript levels of *bdnf* and *narp* are higher when measured after wakefulness (relative to sleep). Learning and plasticity in the neocortex *and* cerebellum, are also both strongly influenced by NA [72, 73]. Learning and plasticity in the cerebellum, however, appear to be governed by LTD, and to a lesser extent, forms of LTP that are quite distinct from those observed in the neocortex [21]. With these caveats in mind, let us consider whether molecular and neurochemical changes conducive for synaptic potentiation also occur during sleep.

There is some molecular evidence for “net” synaptic potentiation during sleep. This includes the observation that nonREM sleep promotes neural protein synthesis—an essential step in persistent forms of synaptic potentiation [74–77]. The complete identities of these proteins are unknown, but some are involved in LTP. For example, a proteomic study in adult rats [77] identified two of these cortical proteins as actin and neuromodulin which play important roles in pre- and postsynaptic modifications, respectively, in LTP [78, 79]. Sleep deprivation also reduces forebrain concentrations of several proteins implicated in LTP (e.g., snap25b, NSF, neuromodulin, neurogranin) [80–83]. The latter experiments suggest that these proteins are normally synthesized during sleep. Collectively, these findings are consistent with net synaptic potentiation as they are based on overall changes within large areas of the brain (e.g., cortical or forebrain)—as opposed to discrete synapses.

Sleep can also promote molecular events conducive for synaptic potentiation within specific regions of the cerebral cortex. During a critical period of development, if vision in one eye is occluded (monocular deprivation: MD), most cortical neurons lose their ability to respond to the deprived eye [84, 85]. This is followed by a strengthening of response to the nondeprived eye [86–88] and anatomical rearrangements of thalamocortical and intracortical circuitry in favor of the intact visual pathway [89, 90]. This form of plasticity, known as ocular dominance plasticity, is considered a canonical model of synaptic plasticity *in vivo* [91, 92].

In the cat, ocular dominance plasticity is consolidated by sleep and this involves synaptic weakening *and *strengthening of cortical circuits [93, 94]. For example, when cortical responses are measured after a period of MD and sleep, responses to the deprived are weaker and responses to the open eye are stronger [94]. The sleep-dependent strengthening of cortical responses is best explained as an increase in glutamatergic synaptic strength. First, consistent with results from LTP protocols, after 1-2 hours of post-MD sleep cortical AMPAR, glur1 subunits are phosphorylated at two sites [94] known to lead to trafficking and insertion of AMPAR in the postsynaptic membrane [95, 96]. The first few hours of sleep are also accompanied by cortical activation of two kinases implicated in LTP at glutamatergic synapses (ERK and CaMKII) [94] and by heightened mTOR-dependent protein synthesis, and increased cortical expression of proteins implicated in LTP (e.g., BDNF and PSD-95) [97]. In rodents, the enhanced response to the open eye is dependent upon Tnf*α*—which promotes glutamatergic synaptic upscaling [98] and is at its highest brain concentrations during sleep [99]. The role of Tnf*α* in feline ODP is unknown, but collectively these findings indicate that the enhanced response to the nondeprived eye involves glutamatergic synaptic potentiation. Interestingly, the effects of MD on nondeprived visual pathways (synaptic potentiation) are retained into adulthood [87, 88]. Given the necessity of sleep for this type of plasticity, this suggests that sleep might have similar effects on cortical circuits throughout the lifespan.

Molecular changes in sleep conducive for synaptic potentiation also occur in adult animals. Sleep-dependent consolidation of two-way active avoidance learning in adult rats is correlated with hippocampal immediate-early gene expression and protein phosphorylation in the first few hours following training [100, 101]. Increased hippocampal expression of the LTP-related gene *zif268 *has been reported in adult rats during rapid eye movement (REM) sleep following exposure to novel, enriched environments [102]. A similar expression of *zif268* is also reported in the hippocampus and cortex during REM sleep following LTP protocols *in vivo* [103]. REM sleep deprivation reduces several molecular markers/mediators of LTP and the ability to induce LTP in the hippocampus; events that are reversed when animals are allowed recovery sleep [104].

The neurochemical milieu of the sleeping brain may also promote synaptic potentiation under certain conditions. REM sleep, for example is well suited for synaptic potentiation as it is characterized by waking levels of membrane depolarization combined with elevated cortical levels of acetylcholine. Indeed, LTP can be reliably induced during this state (reviewed in [4, 5]). Recent studies also show that non-REM sleep can be accompanied by increases in neuromodulators that, according to SHY, mediate synaptic potentiation [105, 106]. Chronic recordings of NA neurons in the principle forebrain source of NA (the locus coeruleus (LC)) show that LC neurons increase their activity during the first 2 hours of post-learning sleep [106]. Intriguingly, the activity of LC neurons is time locked to the cortical slow oscillation of non-REM sleep. LC neurons increase their activity on the rising limb of the cortical upstate [105]; a sequence of events that theoretically could potentiate synapses. An important functional role for such nonREM LC activation is suggested by two recent studies in humans that have shown that manipulating NA during sleep alters olfactory-based, and hippocampal-amygdalar-based learning [107, 108].

## 5. Evidence in Support of SHY

An impressive number of studies provide evidence consistent with SHY. I restrict my discussion to what I consider the most compelling studies. The first are studies in rodents which, in addition to the molecular studies already discussed, report changes in proteins, synaptic efficacy, and dendrite morphology consistent with predictions of SHY [20–22]. Briefly, they show that markers of synaptic potentiation (e.g., changes in AMPAR subunit number or phosphorylation) are elevated in the brains of adult rats sacrificed at the end of the active phase (or after sleep deprivation), relative to animals sacrificed at the end of the rest phase [20]. Similar results are reported for measures of synaptic efficacy (electrically evoked cortical potentials and mEPSCs), which are also elevated at the end of the active phase (or after sleep deprivation) relative to sleep [20, 22]. Two recent imaging studies of cortical dendrite spine morphology showed that the ratio of spines eliminated versus those formed was greater after a period of sleep than a period of wakefulness [21, 109]. Interestingly, these results were restricted to stages of development when there is an overall pruning of synapses and were entirely absent in adult mice [21]. The second are experiments in *Drosophila melanogaster* which show changes in synaptic proteins or morphology consistent with SHY [17–19]. In Drosophila, pre- and postsynaptic proteins and proteins involved in neurotransmitter release are elevated in the brain after extended waking periods or sleep deprivation (relative to sleep) [17]. A second study showed that presynaptic structures, axonal arbors, and postsynaptic spines in Drosophila neurons expanded after extended waking periods (or sleep deprivation); a process also reversed by extended periods of sleep [18]. Similar results were observed in a separate study from Donlea et al. [19].

## 6. The Mechanisms of SHY

The underlying cellular mechanisms governing SHY have not been pursued with equal vigor. Consequently, it is not clear what mechanism, or collection of mechanisms, uniformly explains the synaptic changes reported in insects and mammals. We begin by examining whether those few mechanisms proposed in SHY can explain synaptic changes reported in these species. We then address whether these changes fit with what we know about synaptic scaling, as originally defined and further elaborated by scientists like Turrigiano [7]. This is a reasonable line of inquiry because synaptic scaling features prominently in SHY [9, 10] and is a mechanism for global adjustments of synaptic strength. Lastly, we can consider the role of physiological processes other than sleep that have yet to be excluded as causal factors in the aforementioned findings.

### 6.1. Synaptic Strengthening and Sleep Homeostasis

One important aspect of SHY is that it attempts to link the homeostatic accumulation and discharge of sleep need to synaptic plasticity. According to SHY, synaptic potentiation in wakefulness leads to enhanced sleep need, at least as measured by increased SWA in non-REM sleep. SWA is a reliable index of sleep need in mammals, as it increases in proportion to wake time and decreases during nonREM sleep [14]. A linkage between LTP and SWA is supported by computational models that show that stronger synaptic connections produce higher SWA *in silico *[110, 111]. It has been argued that the neurotrophin BDNF mediates similar events *in vivo *[112, 113]. Brain concentrations of BDNF are highest during waking [30, 31], intracortical infusion of BDNF increases SWA, and intracortical infusion of anti-BDNF antibodies or a BDNF TrkB receptor antagonist decreases SWA [112]. However, it is not clear if these BDNF-mediated changes in SWA are caused by changes in excitatory synaptic strength. While BDNF can promote glutamatergic synaptic potentiation [114], it also promotes GABAergic neurotransmission [62, 115]. Given that GABAergic neurons may also influence SWA [116, 117], it is possible that these results instead reflect changes in inhibitory circuits. The results of intracortical TrkB antagonism are equally difficult to interpret. Removing BDNF (via anti-BDNF antibodies) would affect inhibitory* and* excitatory neurons and the antagonist used (K252A) has non-specific effects on several other kinases [118]. Even if BDNF release during waking leads to net cortical synaptic strengthening, it is not clear why stronger synapses should make one sleepy. Heightened sleep need manifests in several ways, including reduced latencies to sleep and increases in sleep continuity and efficiency. These behavioral aspects of sleep need are not easily explained by simply increasing or decreasing cortical synaptic strength.

### 6.2. SWA and Synaptic Weakening

A principle claim of SHY is that the activity of the sleeping brain—specifically, non-REM SWA—mediates synaptic downscaling [9, 10]. It is suggested, for example, that the periodic appearance of downstates or the frequency of firing during slow oscillations (0.5–4 Hz) could be involved. The idea that SWA promotes synaptic weakening is supported by classic hippocampal LTD protocols, which involve stimulus trains of about 1 Hz. More recent studies *in vitro* and *in situ* indicate that depolarization trains (or intracellular current injections) of about 1 Hz also lead to LTD and the removal of calcium-permeable AMPAR [119, 120], supporting the hypothesis that slow oscillations *in vivo* weaken synapses. While these findings are intriguing, they do not tell the entire story. 1 Hz stimulation that more naturally approximates *in vivo* slow oscillations does not reliably induce LTD in cortical neurons *in situ* [121]. In addition, 1 Hz stimulus protocols that reliably produce LTD *in situ* fail to do so in cortical neurons *in vivo *[122]. These conflicting results may in part reflect limitations inherent in brain preparations *in situ*. These include nonphysiological conditions (e.g., removal of intracortical inhibition and long-range excitatory and neuromodulator inputs), and tissue obtained at ages when sleep regulatory mechanisms are immature [123]. On the other hand, they could also mean that SWA has more than one effect on synaptic strength.

One way that SWA could weaken *or *strengthen synapses is by promoting spike-timing-dependent-plasticity (STDP) [124]. STDP refers to bidirectional changes in synaptic strength that arise from small differences in the timing of presynaptic input relative to postsynaptic depolarization. STDP has been observed under naturalistic conditions in the cortex and the hippocampus *in vivo *[124]. It is thus conceivable that alterations in phase relationship between synaptic inputs and endogenous oscillation during natural brain states (like non-REM sleep) promote synaptic weakening (− phase; postsynaptic firing before presynaptic input) or strengthening (+ phase; pre before post), for additional discussion, see [4, 125]. A strengthening function for SWA is supported by the following findings. First, after learning LC activity precedes the rising edge of cortical upstates, thus providing NA inputs to depolarizing neurons. Second, endogenous busting of neurons within the classic SWA range promotes circuit formation in early life [126, 127] and stimulation protocols that fall within the classic SWA range can also produce LTP [128, 129]. Therefore, the appearance of heightened SWA in sleep after learning [23] (or synaptic potentiation) does not *a priori *mean that synapses are downscaling.

Although SWA theoretically could influence synaptic plasticity, there is little direct evidence for this during natural sleep (for further discussion, see [117]). Computational models show *in silico* that SWA is maximally expressed when cortical synapses are strong and then is reduced when synapses are weakened [110, 111]. One would therefore predict that physiological markers of synaptic potentiation *in vivo* would mirror changes in SWA. For example, if decreases in SWA directly reflect decreases in synaptic strength, then this must involve large, widespread synaptic changes to be detectable at the macrolevel of the EEG. It, therefore, follows that physiological markers of synaptic weakening should be detectable when SWA first declines. In adult rodents, this corresponds approximately to the second or third hour of the rest phase. Most studies, however, measure changes after 6–12 hours of sleep, well after SWA has neared (or obtained) its minima [20, 30]. The few studies that have examined shorter periods of sleep have produced very mixed results [32, 94]. In adult rat frontal cortex, electrically evoked field responses (a measure of cortical potentiation) climb throughout the active phase and modestly decline after the first 2 hours of the rest phase [20]. In the visual cortex, however, evoked field responses progressively decline during the active phase, and increase 2-3 hours after the onset of the rest phase [130]. Interestingly in the latter study, an increase in SWA *preceded* increases in potentiation; findings which are difficult to reconcile with a purely downscaling function for SWA. Similar discrepancies exist for molecular markers of synaptic potentiation. In adult rats, cortical spinophilin—a protein implicated in LTP—is elevated in animals sacrificed 2 hours after the beginning of the rest phase [131]. Another study in adult rats showed that 1 hour of sleep reduces cortical cFOS, but has no effect (relative to wakefulness) on Arc expression [32].

It also appears that SWA cannot be a common mechanism for downscaling in mammals and insects. This is because there is no evidence that the sleeping insect brain displays SWA (or up- and downstates) comparable to birds and mammals. Field recordings in *Drosophila melanogaster* central neurons show that resting states are accompanied by a general reduction of electrophysiological activity [132]. Neural activity in other invertebrate rest states bears little resemblance to mammalian non-REM sleep [133]. In the aquatic invertebrate crayfish, “slow-waves” are reported during reststates, but these waves are not within the typical slow-wave range typical of mammals (>15 Hz).

### 6.3. Synaptic Scaling and SHY

The evidence for SWA-mediated synaptic downscaling is, at best, equivocal, but are other events in the sleeping brain conducive for the global downscaling described by SHY? For example, do the long periods of neuronal silence during downstates, or the neurochemical/molecular changes reported after extended periods of sleep cause synaptic downscaling [9, 10]? We can try to answer these questions by comparing these phenomena with what is actually known about synaptic scaling.

Surprisingly, many of the molecular and electrophysiological findings cited in support of SHY are inconsistent with net synaptic downscaling during sleep (Table 1). The basic principle of synaptic scaling is that decreases in neuronal activity upscale synapses, while increases in neuronal activity downscale synapses (Figure 2). Consequently, downstates in sleep—when vast numbers of cortical neurons are silent—should upscale, not downscale, synapses. Similarly, the neural expression of scaling factors (BDNF, Arc, Homer 1a and Tnf*α*) across the sleep-wake cycle is inconsistent with downscaling during sleep. The low cortical expression of BDNF during mammalian sleep [30–32] should upscale synapses, because reducing BDNF upscales synaptic strength [7, 33]. The low cortical expression of Arc and Homer1a, during sleep [30, 31, 34] should have similar effects because both molecules normally promote synaptic downscaling via AMPAR endocytosis [35–37]. Tnf*α* is released at higher concentrations during sleep [38, 39], has a permissive role in synaptic scaling [134], and promotes synaptic strengthening *in situ* [40] and *in vivo* [98]. Therefore, heightened brain levels of Tnf*α* combined with low levels of Arc, BDNF, and Homer1a during sleep are more conducive for net upscaling rather than net downscaling. With respect to neuromodulators, it has been suggested that sleep-related decreases in the insect analog to NA (octopamine) and NA in mammals might represent a common trigger for downscaling [18]. However, as discussed above, NA has multiple effects on synaptic plasticity and there is no evidence NA must be reduced for downscaling to occur.

An additional unresolved issue is the time course of synaptic scaling and SHY. If downscaling predominantly occurs during sleep, then it follows there is a delay between the induction signal during waking and downscaling. This delay, or arresting of the scaling process, must be hours long (in animals with consolidated wake periods) in order for downscaling to occur in tandem with sleep, but is there any evidence that synaptic scaling must wait for sleep? Although early *in vitro* work indicated that synaptic scaling was a slow process [27], perhaps reflecting a slow accumulation of a scaling signal, more recent studies indicate that scaling can occur very rapidly. At the Drosophila neuromuscular junction, a form of presynaptic scaling can occur within minutes [28]. A rapid form of synaptic scaling is also reported in rodent cortical pyramidal neurons *in vitro* (1 hour) [29]. These studies examined upscaling (not downscaling) and the timecourse of synaptic scaling *in vivo* has not been as finely measured. It is also possible that populations of neurons exhibit “microsleep,” which might promote more rapid scaling [135]. Nevertheless, these studies suggest that downscaling might occur concurrently with Hebbian plasticity and without sleep.

## 7. Alternative Mechanisms

Given the uncertainty surrounding SHY mechanisms, it is important to consider physiological processes other than sleep that have yet to be excluded as factors. These include changes in brain temperature and glucocorticoids (i.e., corticosterone/cortisol). Both brain temperature and glucocorticoid release are strongly regulated by the circadian system. Brain temperature and glucocorticoid release are maximal during the active phase, both reach their nadir during the rest phase, but both are elevated by sleep deprivation (in rodents) [136–140]. Temperature and glucocorticoids also modulate neuronal function and synaptic efficacy in ways that resemble changes reported as evidence of SHY. Consequently, differences in synaptic efficacy or proteins analyzed at different circadian times may be due to differences in brain temperature or glucocorticoids, rather than vigilance state. The normal control for circadian effects (i.e., sleep deprivation in the rest phase) may be inadequate, as sleep deprivation increases brain temperature and glucocorticoids in rodents.

### 7.1. Brain Temperature

A role for brain temperature is suggested by the fact that the most dramatic evidence of SHY is found in ectothermic insects [17–19]. In contrast to birds and mammals, ectotherms do not internally regulate their core/brain temperature. Temperature is instead behaviorally regulated, either by selecting warmer environments or through activity [141]. As discussed above, long periods of sleep in *Drosophila melanogaster* massively prune back synaptic proteins and structures [17–19]. In rodents, changes in similar structures (e.g., dendritic spines) after sleep are much more modest and restricted to a narrow window of development [21, 109]. Interestingly, the only mammals which display large-scale synaptic changes during sleep-like states comparable to *Drosophila melanogaster* are hibernators [142, 143]. During hibernation (which is entered through sleep), brain temperature precipitously declines and there is a massive retraction of dendrites and synapses. This is followed by a rapid expansion of these structures during arousal and euthermia [142, 143]. These changes are strikingly similar to changes reported in sleep and wake in *Drosophila melanogaster* [17–19]. This raises the possibility that the results reported in ectotherms are not related to wakefulness or sleep *per se*, but to accompanying changes in core/brain temperature. Indeed, warm ambient temperatures lead to several changes in adult and larval Drosophila neurons that resemble those reported after long periods of wake (relative to sleep). These include increased axonal arborization in mushroom body neurons [144] and motor nerve terminals *in vivo* [145] and neurite extension *in vitro* [144]. Intriguingly, these temperature effects are mediated by signaling pathways shared by activity-dependent synaptic plasticity (e.g., cAMP) [144]. Whether similar temperature gradients exist across insect wake and sleep is unknown as this has yet to be measured. However, given that core temperature tracks motor activity in small terrestrial insects [141], sleep and wake may be accompanied by significant changes in brain temperature. A strong temperature effect in terrestrial insects may also explain the very faint effects of sleep on synaptic proteins in the zebrafish *Danio rerio* [146]. Zebrafish are also ectotherms, but are well adapted to fluctuations in surrounding temperature [147] and unlikely to experience large temperature gradients under experimental conditions [146].

Strong temperature effects in endothermic mammalian neurons are also reported under certain conditions. Hippocampal dendritic spines *in situ* are highly sensitive to changes in temperature, rapidly shrinking then reexpanding with cooler and warmer temperatures [148]. Similar temperature effects are observed in proteins that make up the postsynaptic density [148]. Cooling the hippocampus *in situ* reversibly reduces excitatory postsynaptic field potentials (EPSPs), and reverses (de-potentiates) LTP [149]. Conversely, transient warming of hippocampal slices has biphasic effects, an initial depression, then prolonged enhancement of EPSPs [150]. Temperature effects are not restricted to the hippocampus, as the rate of mEPSCs in rodent cortical neurons is surprisingly temperature sensitive (Q10 of 8.9) [151]. One must be cautious in extrapolating from studies *in situ *or* in vitro*, which use large temperature gradients, to the situation *in vivo*. However, strong effects of naturally occurring brain temperature gradients on EPSPs are reported in freely behaving rodents [152]. As shown by Moser et al. [152], motor activity increases hippocampal temperature and EPSPs. This EPSP enhancement is unrelated to learning-related plasticity. It is instead caused by the normal rise in brain temperature associated with waking movement and dissipates as the brain naturally cools.

### 7.2. Glucocorticoids

In rodents, corticosterone also rises and falls in parallel with wake and sleep and has profound effects on synaptic efficacy and plasticity molecules. As is true for neuromodulators like NA (which is activated synergistically with corticosterone), these effects are diverse and dependent upon different classes of receptors [153]. They have also been chiefly explored in the hippocampus rather than the neocortex. Nevertheless, circadian increases in corticosterone (i.e., during the normal waking period), or after sleep deprivation may generally promote glutamatergic neurotransmission and neuronal excitability (relative to sleep) [153]. Acute increases in corticosterone (or stress) increase the frequency [154] and amplitude of mEPSCs in the hippocampus [155], strengthen glutamatergic synapses onto dopamine neurons [156], and increase glutamatergic release/calcium mobilization in cortical synaptoneurosomes [157]. Acute increases in corticosterone also promote AMPAR synaptic transmission, AMPAR trafficking and insertion into cortical and hippocampal synapses, and cortical dendritic spine turnover [158–161].

In conjunction with circadian rhythms in brain temperature, the cumulative effects of increased (wake) or decreased (sleep) corticosterone release may explain a number of findings in rodents ascribed by SHY to sleep and wake. These include differences in evoked field potentials [20] and mEPSCs [22] in animals examined at circadian times of low and high corticosterone release (or after sleep deprivation). They might also contribute to relative (sleep versus wake) differences in synaptic proteins, plasticity molecules, and dendritic spine morphology obtained from rodents sacrificed after long periods of waking and sleep [20, 21, 31]. A strong circadian component to SHY may also explain why mammals with weak circadian rhythms [94] do not show the same sleep-related decreases in “LTP” molecules and AMPAR phosphorylation as rodents [20].

## 8. Discussion

A scientific theory can be evaluated by several criteria. Does it attempt to explain and predict, better than other theories, empirical findings? Does it stimulate other scientists to challenge prior assumptions and perform new experiments? Does it address a problem of broad scientific interest and importance? In many new and exciting ways, SHY satisfies these criteria and thus represents a valuable contribution to the study of sleep, but, as with any new scientific theory, much more work is needed before its true importance can be gauged.

The core theoretical concepts of SHY are puzzling in several respects. The idea that waking and sleep are dominated by net synaptic potentiation and weakening, respectively, requires a very narrow view of brain plasticity. The waking brain is typified by many forms of learning, each likely employing complex combinations of Hebbian and non-Hebbian plasticity. If these waking forms of plasticity require secondary sleep-dependent processes, it is not clear why the latter should primarily manifest as (or sum to) “net” synaptic weakening. What seems more likely is that sleep is characterized by multiple forms of synaptic plasticity, including classic Hebbian LTP and LTD [4], as well as downscaling *and* upscaling. This may explain why the evidence for “net” downscaling after sleep critically depends on what is measured (e.g., neuromodulin versus BDNF) and when those measurements are made (e.g., early or late in the rest phase). What determines the types of plasticity engaged during sleep is unknown, but in addition to waking experience ontogenetic factors are likely important. For example, sleep amounts are maximal during periods of heightened synaptogenesis including *in utero* when waking experience is negligible [162, 163]. It seems highly unlikely that a fundamental purpose of sleep is to principally weaken synapses during these developmental periods.

A second unresolved issue is the function of sleep-dependent downscaling. It is theorized that downscaling in sleep improves signal-to-noise, which would benefit memory consolidation, or allow for new learning to occur during subsequent waking [9, 10]. This is because, according to SHY, functional synapses are preserved while nonfunctional ones are eliminated. Indeed, from a purely theoretical view, such precise scaling during sleep would be highly adaptive. Computational models support this idea [111], but this is largely untested *in vivo*. There is no evidence that the changes in neural protein phosphorylation [20], Arc or BDNF [30, 31], or dendrite morphology [164] reported after rodent sleep contribute to cognition or other adaptive behavior. In *Drosophila melanogaster*, sleep is required for new learning to occur, and this sleep is accompanied by a reduction in synapses [19]. However, it is unknown if this change in synapses and not some other process during sleep is the causal factor.

One important future direction is to delve more deeply into the underlying mechanisms of SHY. To date, this has received less attention than studies aimed at collecting supportive findings. As a consequence, it is not clear if the observed phenomena are due to sleep *per se*, or other physiological processes that coincide with sleep. One way to address this issue is to design experiments that address these factors. For example, does brain cooling in insects replicate (and brain warming prevent) the effects of sleep on neuronal morphology and synapses? Do the same changes in mammalian synaptic efficacy, proteins, and dendrites observed after wake and sleep occur when corticosterone is experimentally clamped? This can be accomplished with adrenalectomy combined with hormone replacement—as recently demonstrated by Mongrain et al. [138]. A second way is to perform strong tests of SHY using modern molecular tools *in vivo*. According to SHY, cortical synaptic potentiation in wakefulness is a causal factor in sleep homeostasis (as measured by SWA) [112]. Therefore, one would predict that transgenic mice with deficits in cortical synaptic potentiation should also show reductions in sleep need. It would be interesting, for example, to examine sleep homeostasis in (CaM) KII^T286A^ point mutation [96, 165] and PKA RII*α* null mutant (−/−) mice [166], which exhibit large reductions in cortical LTP. There are also several techniques for inducible (as opposed to constitutive) deletion of floxed genes—which would allow one to examine sleep homeostasis after deletion of molecules necessary for cortical LTP (e.g., BDNF). Third, given that mechanisms involved in synaptic scaling are increasingly well understood, what is their expression pattern in the awake and sleeping brain? If their expression pattern is inconsistent with SHY, which so far appears to be the case, then what specific plasticity mechanism is responsible? Some promising work comes from Lanté et al., who showed that AMPAR internalization *in vitro* involves phosphatase and protein kinase C activity [119]. Do these signaling pathways play an equally important role in sleep-mediated plasticity *in vivo?*

A second important future direction is to integrate synaptic changes related to SHY with other sleep-dependent forms of plasticity. As discussed above, plasticity in the visual cortex is consolidated by sleep and this includes changes that are best explained by synaptic potentiation [94]. Sleep in mammals is also accompanied by hippocampal bursts of activity that “replay” patterns present during experience. This replay occurs during high-frequency firing (“ripples” and “sharp waves”) that are well suited for events like LTP [167]. Thalamocortical spindles may also mediate various forms of synaptic strengthening during mammalian sleep [168, 169]. In *Drosophila melanogaster*, sleep not only scales back synapses presumably allowing new learning to occur, but sleep *after* learning is needed to make long-term memory; a process that requires the formation of new synapses [19]. In developing mice, cortical dendritic spines are not only eliminated, but also formed during sleep [21, 109]. Therefore, it is conceivable that sleep promotes a generalized synaptic downscaling, accompanied with Hebbian or non-Hebbian synaptic potentiation in select circuits [170–172]. However, this presupposes that net downscaling is directly sleep-dependent and as discussed above, this has not been conclusively shown.

## 9. Concluding Remarks

Over the last 100 years, numerous grand or unifying theories of sleep function have been proposed [16]. None, however, have adequately explained the presence of sleep across the animal kingdom, its unusual electrophysiological, neurochemical and molecular events, and its dramatic changes across the lifespan [16]. As new experiments accumulated, their predictive power failed, and they became little theories that only explained—often imperfectly—single sleep phenomena [16]. It is too soon to say where SHY fits in this story. SHY is a seminal theory, bold in its scope and challenging in its implications, but it seems oddly disconnected from our rapidly evolving views of synaptic plasticity. The proponents of SHY have also amassed an impressive set of supportive findings, but these have yet to be pursued in depth. These are not trivial matters. In the absence of a clearly proposed mechanism (informed by current views on synaptic plasticity), the empirical supports of SHY are hard to interpret. Therefore, the significance of SHY—and what it may one day reveal about sleep and synaptic plasticity—remains elusive.

## Figures and Tables

**Table 1:** Scaling factors and the sleep-wake cycle.

	Wake	Sleep	Promotes
BDNF	↑↑	↓↓	Synaptic downscaling [7, 30–33]
Arc	↑↑	↓↓	Synaptic downscaling [30, 31, 34–37]
Homer1A	↑↑	↓↓	Synaptic downscaling [34, 37]
Tnf*α*	↓↓	↑↑	Synaptic upscaling [38–40]
Retinoic acid	??	??	Synaptic upscaling* [41, 42]

The expression of scaling factors is inconsistent with net downscaling during sleep.

*Retinoic acid is linked to SWA generation, but sleep/wake expression patterns are unknown.

## Abbreviations

AMPAR: *α*-amino-3-hydroxy-5-methyl-4-isoxazolepropionic acid receptor
Arc: Activity-regulated cytoskeleton-associated protein
BDNF: Brain derived neurotrophic factor
CAMK: Calmodulin-dependent kinase
EPSP: Excitatory post-synaptic potential
ERK: Extracellular regulated kinase
GABA: Gamma-aminobutyric acid
LC: Locus coeruleus
LTD: Long-term depression
LTP: Long-term potentiation
mEPSC: Miniature excitatory post-synaptic current
NA: Noradrenaline
Narp: Neuronal activity-regulated pentraxin
NMDA: n-methyl-d-aspartic acid
NSF: N-ethylmaleimide-sensitive factor
PKA: Protein kinase A
Q10: The rate of change of a biological or chemical system following a 10°C change in temperature
REM: Rapid eye movement
SHY: Synaptic homeostasis hypothesis
Snap25b: Synaptosomal-associated protein, 25 k
STDP: Spike-timing dependent plasticity
SWA: Slow wave activity
Tnf*α*: Tumor necrosis factor-alpha
TrkB: Tyrosine receptor kinase B
zif268: Zinc finger protein 225 or NGFI-A (nerve growth factor-induced protein A).
